# Effect of acute noise trauma on the gene expression profile of the hippocampus

**DOI:** 10.1186/s12868-020-00599-9

**Published:** 2020-11-07

**Authors:** Chang Ho Lee, Kyung Woon Kim, So Min Lee, So Young Kim

**Affiliations:** Department of Otorhinolaryngology, CHA University College of Medicine, 59 Yatap-ro, Bundang-gu, Seongnam, 13496 Gyeonggi-do Korea

**Keywords:** Hippocampus, Noise, Hearing loss, Microarray analysis, Genetic association studies

## Abstract

**Background:**

This study aimed to investigate the changes in the expression of hippocampal genes upon acute noise exposure.

**Methods:**

Three-week-old Sprague–Dawley rats were assigned to control (n = 15) and noise (n = 15) groups. White noise (2–20 kHz, 115 dB sound pressure level [SPL]) was delivered for 4 h per day for 3 days to the noise group. All rats were sacrificed on the last day of noise exposure, and gene expression in the hippocampus was analyzed using a microarray. Pathway analyses were conducted for genes that showed differential expression ≥ 1.5-fold and *P* ≤ 0.05 compared to the control group. The genes included in the putative pathways were measured using quantitative reverse transcription-polymerase chain reaction (qRT-PCR).

**Results:**

Thirty-eight upregulated genes and 81 downregulated genes were identified. The pathway analyses revealed that upregulated genes were involved in the cellular responses to external stimuli and immune system pathways. qRT-PCR confirmed the upregulation of the involved genes. The downregulated genes were involved in neuronal systems and synapse-related pathways, and qRT-PCR confirmed the downregulation of the involved genes.

**Conclusions:**

Acute noise exposure upregulated the expression of immune-related genes and downregulated the expression of neurotransmission-related genes in the hippocampus.

## Background

Hearing loss has been suggested to be associated with cognitive deficits [1]. Although conflicting data have been reported for the bidirectional association or the weak contribution of peripheral hearing loss to cognitive dysfunction [2, 3], many clinical studies have confirmed the effect of peripheral hearing loss on cognitive dysfunction [1]. Auditory sensory deficits and accompanying disabilities, such as communication problems and social isolation, may alter the hippocampus and result in cognitive dysfunction in patients with chronic hearing loss.

Intense noise exposure is known to cause permanent hearing loss and stress responses [4–6]. An increasing number of experimental studies have supported the association between noise exposure and cognitive dysfunction [7–9]. A study of a senescence-prone mouse model revealed that chronic noise exposure for 30 days reduced the activity of the Wnt signaling pathway and increased amyloid-beta accumulation and tau hyperphosphorylation in the hippocampus [9]. Similarly, a study of Wistar rats reported tau hyperphosphorylation and increased corticotropin-releasing factor levels after 30 days of noise exposure [7]. Although most previous studies have examined the effects of long-term noise exposure on cognitive function, a shorter duration of noise exposure for 15 days also resulted in behavioral changes and neurotransmitter changes in the hippocampus [8]. In addition, abnormal connectivity and neural activity were observed in the hippocampus after 1 day of noise exposure [10]. Another mouse study reported that a single 2-h noise exposure induced behavioral deficits and increased thioredoxin levels in postnatal day 7–15 mice [11]. Thus, acute and chronic noise exposure may be implicated in hippocampal changes related to both hearing loss and stress stimuli.

The current study hypothesized that acute noise exposure might alter gene expression in the hippocampus. By identifying altered genes, early changes in the hippocampus can be revealed and biomarkers or therapeutics can be discovered. The gene expression profile of the hippocampus was evaluated using microarray analyses immediately after exposure to intense noise stimuli to investigate early changes in the hippocampus following noise exposure, which might be attributed to stress stimuli and result in permanent hearing loss. We used 3-week-old rats to exclude the effects of aging.

## Methods

### Animal groups and noise exposure

The Institutional Animal Care and Use Committee of CHA University Medical School (IACUC190046) approved the experiments described in the present study. All experiments complied with the guidelines of the Institutional Animal Care and Use Committee of CHA University Medical School. Postnatal day 21 female Sprague–Dawley rats were used.

**Fig. 1:**
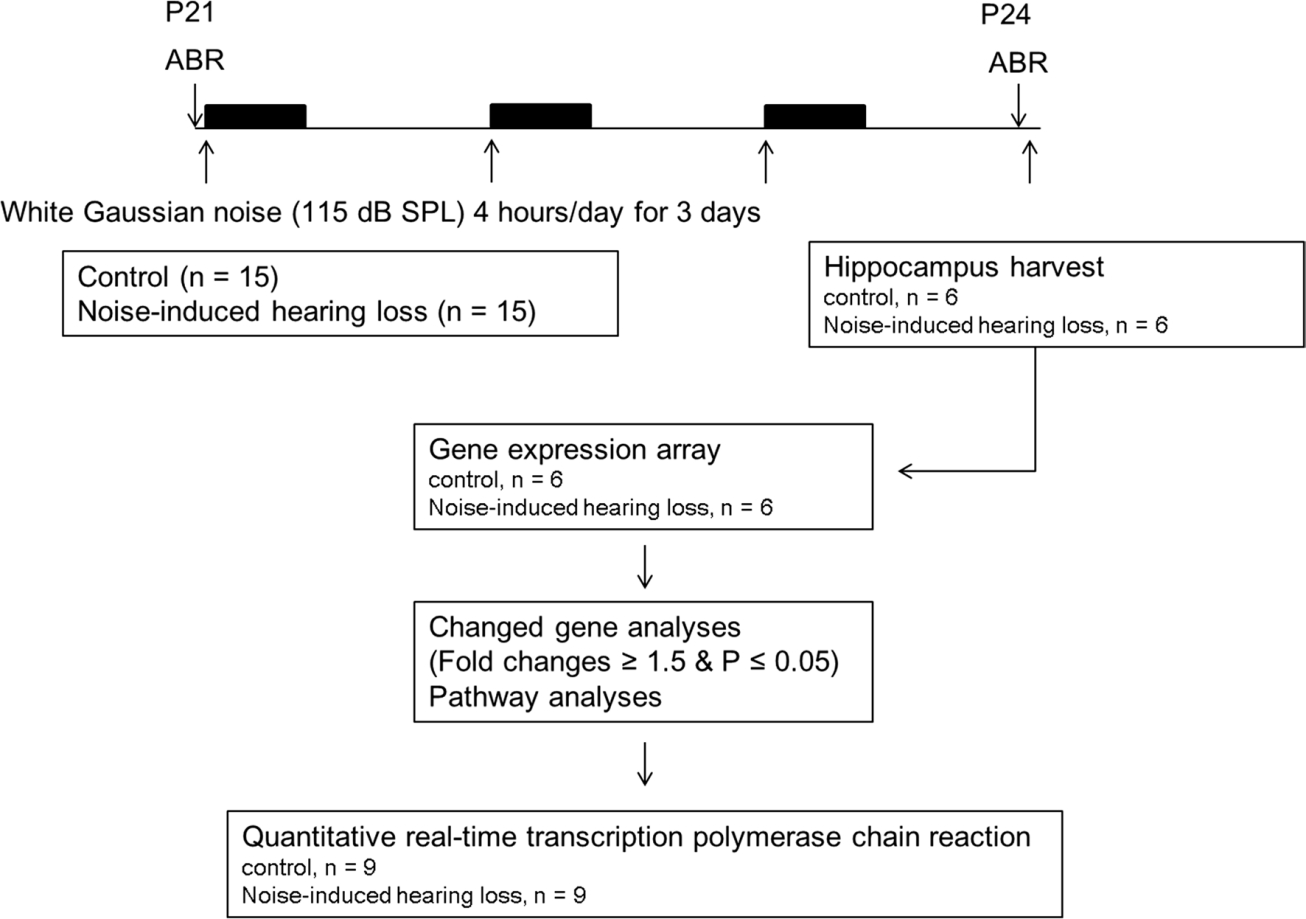
The experimental schedule of the present study. Three-week-old rats were divided into the control (n = 15) group or the noise group (n = 15). White noise was delivered for 3 days. The hippocampus was harvested from 12 rats (n = 6 rats per group) and gene expression was analyzed using a microarray. Pathway analyses were performed for the DE genes and qRT-PCR was conducted using samples from 18 rats (n = 9 rats per group)

Thirty rats were divided into control (n = 15) and noise (n = 15) groups (Fig. 1). Rats in the noise group were exposed to 2–20 kHz, 115 dB SPL white noise for 4 h/day for 3 consecutive days. Noise was provided throughout a sound chamber via a free-field electrostatic speaker (Tucker-Davis Technologies, Alachua, FL, USA), which was located on top of the chamber. Rats remained awake during noise exposure. The control group was not exposed to noise. The control group was placed in an identical chamber to the noise group, but were not exposed to noise and were subjected to a background noise of approximately 40 – 60 dB SPL for the same duration. Both the noise and control groups were housed under identical standard conditions.

### Auditory threshold measures

**Fig. 2:**
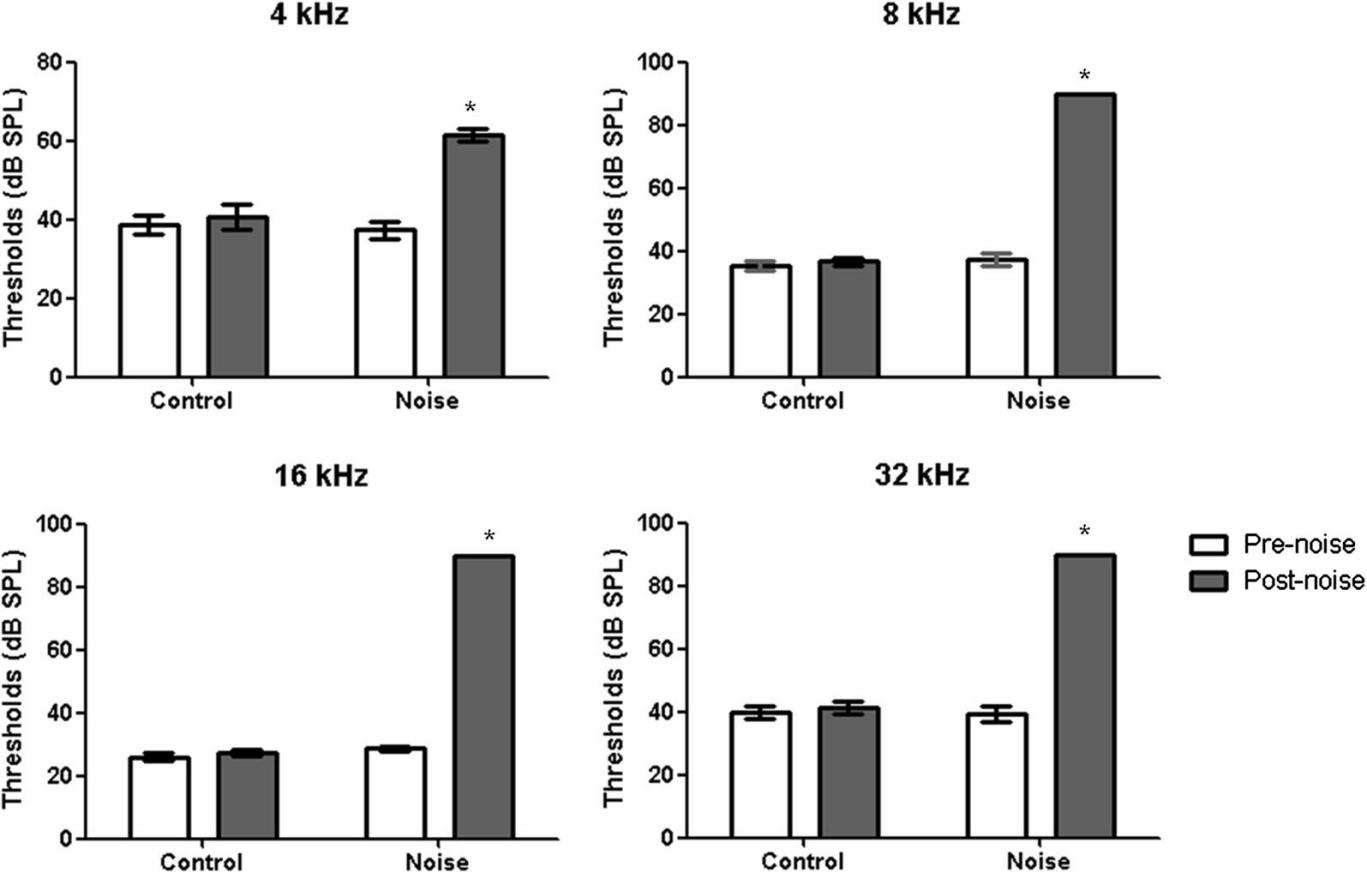
The average thresholds of the ABR after noise exposure. The average ABR thresholds at 4, 8, 16, and 32 kHz were increased in the noise group (all *P* < 0.001, independent sample *T*-test comparing post-control and post-noise)

Immediately after the 3 days of noise exposure, hearing thresholds in all rats were measured by recording auditory brainstem responses (ABRs) (SmartEP; Intelligent Hearing System, Miami, FL, USA) as previously described [12, 13] (Fig. 2). After rats were anesthetized using a mixture of Zoletil (40 mg/kg) and xylazine (10 mg/kg), subdermal needle electrodes were inserted into the vertex, behind the ipsilateral pinna, and behind the contralateral pinna as a ground electrode [14]. A plastic earphone was applied to the external auditory canal and connected to an EC1 electrostatic speaker. Tone bursts (duration, 1562 µs; envelope, Blackman; stimulation rate, 21.1/s) at 4, 8, 16 and 32 kHz were delivered. The amplified evoked responses with 1,024 sweeps were averaged. The filter settings were 300 Hz on the high-pass end and 3000 Hz on the low-pass end. The initial sound intensity was 80 dB SPL and was reduced at 10 dB SPL intervals. The auditory threshold was defined as the lowest sound intensity that evoked wave II [15, 16]. If an ABR was not detected at 80 dB SPL, the hearing threshold was reported as 90 dB SPL.

### Microarray analyses

Six to eight hours after the ABR measurement, all rats were sacrificed and their hippocampi (n = 15 per group) and primary temporal cortices (n = 6 per group) were harvested. The euthanasia was conducted by carbon dioxide inhalation with flow rate of 5–6 L/min in 25.40 ×48.26 ×22.86 cm size cage, as previously described [17]. The dorsal cornu ammonis 3 (CA3) region of the rat hippocampus was located in a reference atlas, and identical tissue samples were dissected from adjacent regions of CA2 and the dentate gyrus [18]). The hippocampi from 12 rats (6 rats per group) were used for the microarray analysis, and the tissues from 18 rats (9 rats per group) were used for the real-time quantitative reverse transcription-polymerase chain reaction (qRT-PCR) analysis.

RNA was extracted from the hippocampal tissues using TRI reagent® (Sigma-Aldrich, St. Louis, MO, USA) [12, 13]. Two hippocampal tissue samples were pooled as a single sample for the total transcript array analysis to increase the quantity of the extracted RNA. The purified RNA was assessed for purity and quantity by measuring the ratio of the absorbance at 260/280 nm using a NanoDrop^TM^1000 spectrophotometer (Thermo Scientific, Madison, WI, USA). Only samples with a 260/280 nm ratio greater than 1.8 and a 260/230 nm ratio greater than 1.5 were eligible for inclusion in the microarray analysis. The quality of the RNA was assessed using an Agilent 2100 Bioanalyzer™ (Agilent Technologies, Santa Clara, CA, USA) ('Genomics Agilent') [19]. Only samples with an RNA Integrity Number greater than 7.0 were eligible for inclusion in the microarray analysis. No samples were excluded due to a low RNA quality. Five hundred nanograms of total RNA were used to generate cDNAs with a GeneChip WT PLUS Reagent Kit (Affymetrix Inc., Santa Clara, CA, USA). Samples were amplified using the WT amplification reagents from the GeneChip WT PLUS Kit. Following amplification, cDNA samples were purified using purification beads. Sample concentrations were determined using a 33 μg/ml/A260 constant on a NanoDrop1000 spectrophotometer. Exactly 5.5 μg of ss-cDNA were fragmented by uracil-DNA glycosylase (UDG) and apurinic/apyrimidinic endonuclease I (APE I) and labeled with terminal deoxynucleotidyl transferase (TDT) using the proprietary Affymetrix DNA labeling reagent.

The total mRNA transcripts from 6 samples (3 samples from each group, each sample was a pool of 2 hippocampal tissues, for a total of 12 hippocampi) were analyzed using the Affymetrix Rat Gene ST 2.0 array at BioCore Co., Ltd. (Seoul, South Korea). A hybridization control mixture containing B2 Control Oligo, 20 X hybridization controls (bioB, bioC, bioD, and cre), DMSO, 2 X hybridization buffer and water was added to all six samples. One hundred nine microliters of this mixture were injected into a GeneChip Rat Gene 2.0 ST Array (Affymetrix, Santa Clara, CA, USA) and placed in the Affymetrix GeneChip Hybridization Oven 640 at 45 °C and 60 rpm for 16 h. Stain cocktails (stain cocktail 1 and stain cocktail 2 [GeneChip Hybridization, Wash, and Stain Kit]) were added to amplify the signal intensities. The arrays were stained and washed in an Affymetrix GeneChip Fluidics Station 450 according to the FS450_0002 fluidics protocol. All arrays were scanned with the Affymetrix GeneChip Scanner 3000, and raw data were analyzed with Transcriptome Analysis Console™ (TAC) software. The raw data images, which contained reference intensities for each probe on the array, produced by the scanner were processed into CEL files.

The CEL files were imported into the Gene Expression Workflow in GeneSpring GX version 14.9.1 (Agilent Technologies Inc.). The Robust Multi-Array Average (RMA) algorithm (for background correction, log2 transformation, and probe set summarization) with the default settings was used in the GeneSpring software. Genes that were differentially expressed (DE) between the noise and control groups were predicted (probe sets were summarized into transcript clusters/genes). A principal component analysis (PCA), which reduces the dimensionality of a dataset with a large number of interrelated variables, was performed using a covariance dispersion matrix for data quality control (Fig. S1).

### Statistical analysis of microarray expression data

Independent sample T-tests were used to compare the expression of individual genes between the noise group and the control group. Differential expression was defined as an absolute fold change ≥ 1.5 and a *P* value ≤ 0.05. The input for the heatmap was the log2-transformed DE genes with an absolute fold change ≥ 1.5 and a *P* value ≤ 0.05. For these transcripts, overrepresentation analyses were conducted using ConsensusPathDB (cpdb.molgen.mpg.de). All genes measured in the microarray were provided as a list of background genes. The associated pathways were identified with Reactome and the Kyoto Encyclopedia of Genes and Genomes. Two or more involved genes and *P* < 0.05 were used as the criteria for the associated pathways. The *P* values were calculated using the hypergeometric test based on the number of physical entities present in both the predefined set and specified list of physical entities. The *P* values were corrected for multiple testing using the false discovery rate method and are presented as Q values. Only pathways related to hippocampal function were listed as the final related pathways.

### Confirmation of gene expression levels using qRT-PCR

**Fig. 3:**
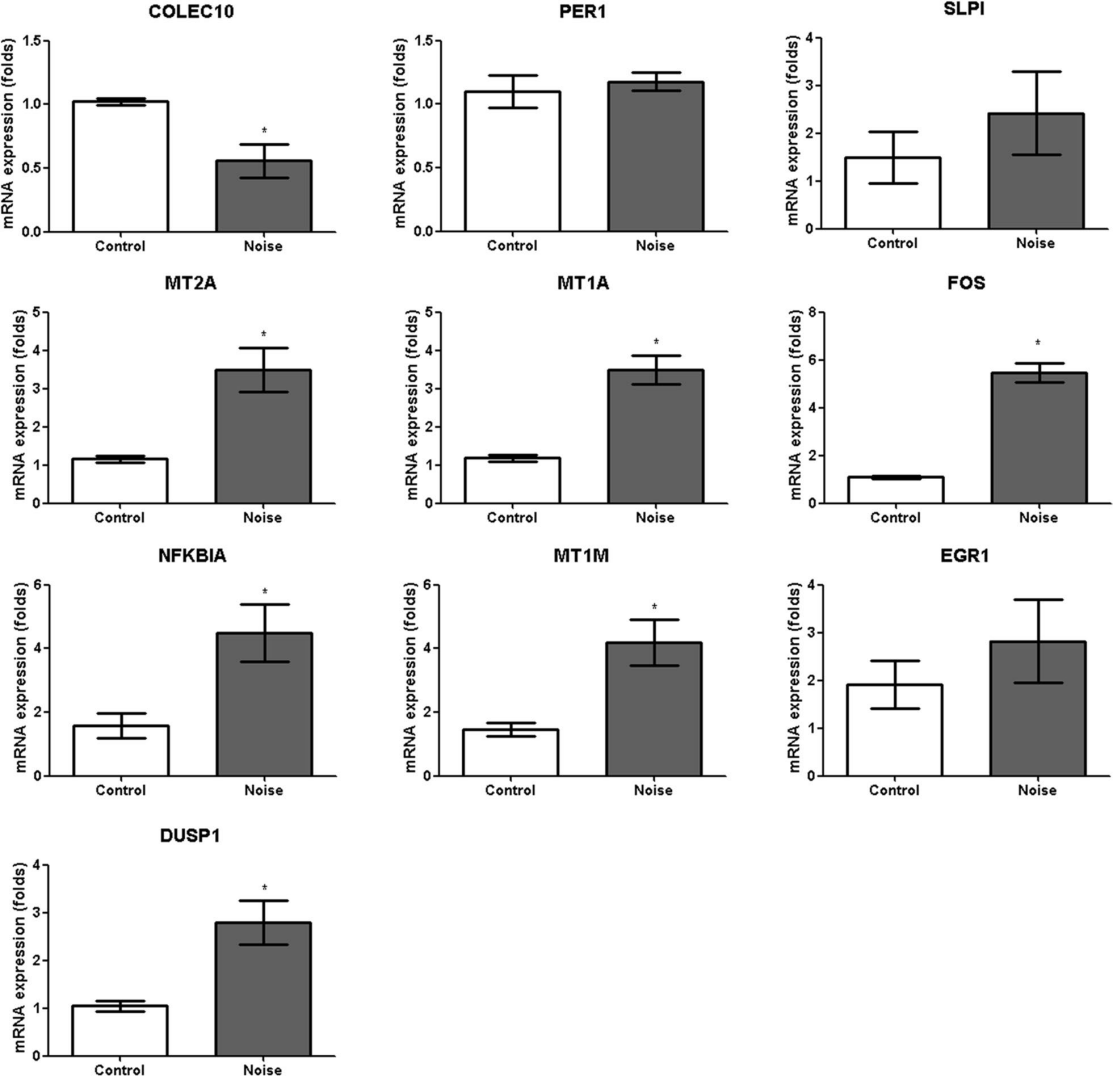
The mRNA expression levels measured using qRT-PCR. The expression of the *MT2A, MT1A, FOS, NFKBIA, MT1M*, and *DUSP1* mRNAs was increased in the noise group compared to the control group (3.00-fold (3.49/1.16) [SD = 0.58], *P* < 0.001 for *MT2A*, 2.96-fold (3.49/1.18) [SD = 0.58], *P* < 0.001 for *MT1A*, 5.01-fold (5.46/1.09) [SD = 0.41], *P* < 0.001 for *FOS*, 2.82-fold (4.48/1.59) [SD = 0.91], *P* = 0.006 for *NFKBIA*, 2.88-fold (4.20/1.46) [SD = 0.73], *P* = 0.002 for *MT1M*, and 2.66-fold (2.79/1.05) [SD = 0.46], P = 0.008 for *DUSP1*, Mann–Whitney *U* test). The levels of the *COLEC10, PER1, SLPI*, and *EGR1* mRNAs were not significantly different in the noise group compared to the control group (0.56-fold (0.56/1.00) [SD = 0.13], *P* = 0.006 for *COLEC10*, 1.17-fold (1.17/1.00) [SD = 0.07], *P* = 0.61 for *PER1*, 1.62-fold (2.42/1.49) [SD = 0.0.87], *P* = 0.60 for *SLPI*, and 1.48-fold (2.82/1.91) [SD = 0.87], P = 0.43 for *EGR1*, Mann–Whitney *U* test)

**Fig. 4:**
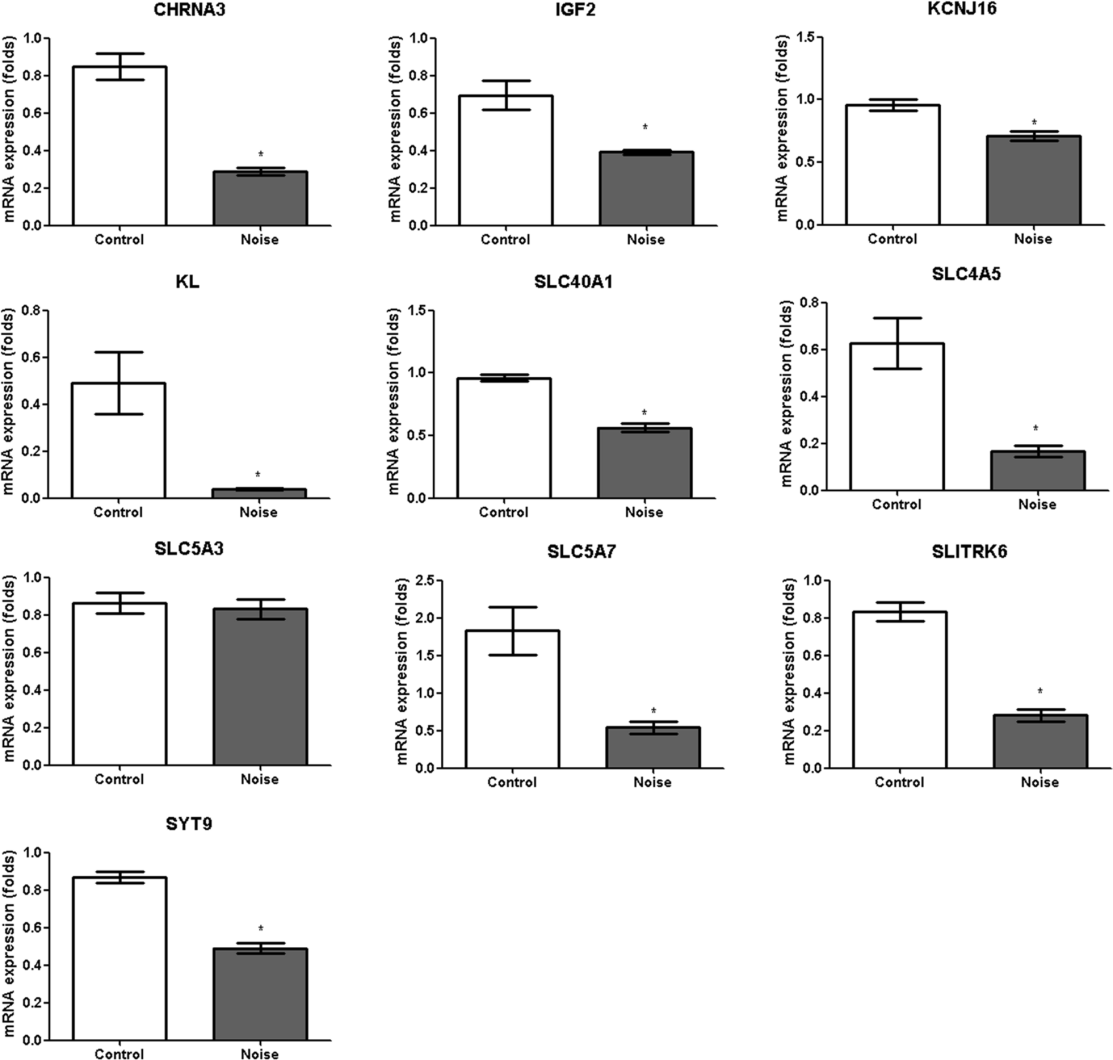
The mRNA expression levels measured using qRT-PCR. The levels of the *CHRNA3, IGF2, KCNJ16, KL, SLC40A1, SLC4A5, SLC5A7, SLITRK6,* and *SYT9* mRNAs were decreased in the noise group compared to the control group. (*CHRNA3* (0.34-fold (0.29/0.85) [SD = 0.02]), *IGF2* (0.57-fold (0.39/0.69) [SD = 0.01]), *KCNKJ16* (0.74-fold (0.71/0.96) [SD = 0.03]), *KL* (0.08-fold (0.49/0.04) [SD = 0.01]), *SLC40A1* (0.58-fold (0.56/0.96) [SD = 0.10]), *SLC4A5* (0.27-fold (0.17/0.63) [SD = 0.02]), *SLC5A7* (0.30-fold (0.54/1.83) [SD = 0.24]), *SLITRK6* (0.34-fold (0.28/0.83) [SD = 0.03]), and *SYT9* (0.56-fold (0.49/0.87) [SD = 0.02]), Mann–Whitney *U* test)

**Fig. 5:**
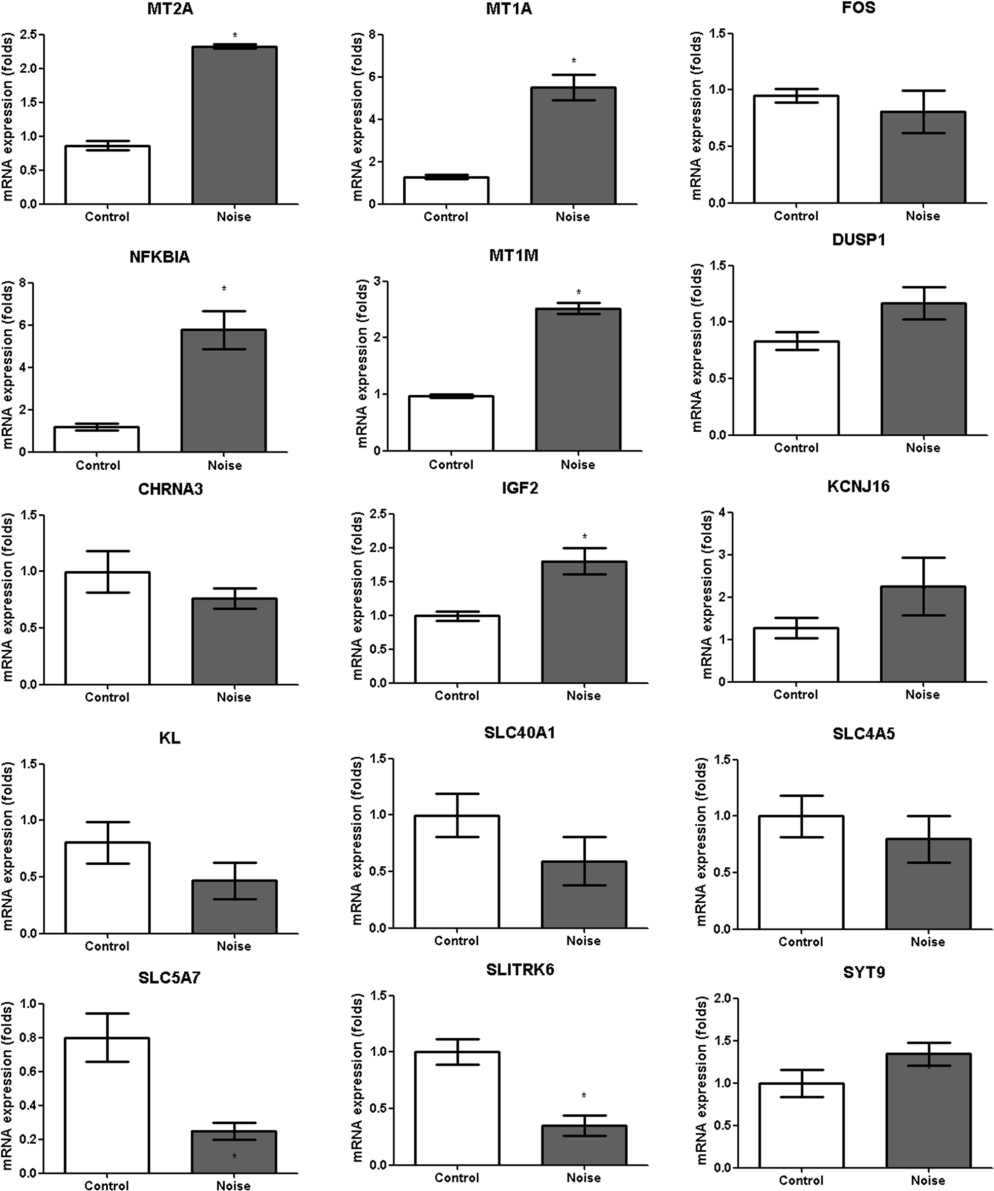
The mRNA expression levels in the temporal cortex measured using qRT-PCR. The levels of the *MT2A* (2.69-fold (2.32/0.86) [SD = 0.03], *P* = 0.002), *MT1A* (4.30-fold (5.50/1.28) [SD = 0.61], *P* = 0.002), *MT1M* (2.60-fold (2.52/0.97) [SD = 0.09], *P* = 0.002), and *NFKBIA* (4.89-fold (5.77/1.18)[SD = 0.91], *P* = 0.002) mRNAs were increased the temporal cortex of the noise group. Regarding the genes that were downregulated in the hippocampus, the levels of the *SLC5A7* (0.31-fold (0.25/0.80) [SD = 0.05], *P* = 0.004) and *SLITRK6* (0.35-fold (0.35/1.00) [SD = 0.09], *P* = 0.004) mRNAs were decreased in the temporal cortex of the noise group

Nine rats in each group were used for the qRT-PCR studies. qRT-PCR was performed for the DE genes involved in the altered pathways, as previously described [12, 13]. qRT-PCR was conducted according to the relevant guideline [20]. RT-PCR was performed using a ViiA7 Real-time PCR system (Applied Biosystem, Carlsbad, CA, USA) with TOPreal™ qPCR 2 × PreMIX (SYBR Green with low ROX; Enzynomics, Daejeon, Korea) and the following protocol: initial activation of HotStarTaq® DNA polymerase at 95 °C for 15 min followed by 50 cycles of 95 °C for 10 s, 60 °C for 15 s, and 72 °C for 15 s. The amplification efficiency (E) of each amplicon was determined using tenfold serial dilutions of a positive control complementary DNA (cDNA) and calculated from the slopes of the log input amounts (from 20 ng to 2 pg of cDNA), which were plotted according to the crossing point values using the formula E = 10^–1/slope^. The forward and reverse primers are listed in Table 1. All primer efficiencies were confirmed to be high (> 90%) and comparable. The calculated mRNA levels were normalized to the glyceraldehyde 3-phosphate dehydrogenase mRNA according to the formula 2^–Ct^, and expressed as a percentage of the reference gene. The gene expression levels of control rats in group 1 were estimated as the reference level of gene expression as presented in graphs of Figs. 3,4, and 5. The fold changes in gene expression levels of the noise group based on those of control group (mean of control group) were calculated. The upregulated and downregulated genes in the hippocampus were evaluated for changes in mRNA expression in the primary auditory cortex (n = 6 per group) to compare the noise exposure-induced changes in gene expression between the hippocampus and primary auditory cortex. The primary auditory cortex was localized according to the coordinates in the Paxinos and Watson atlas (A/P = -2.7 to 5.8 mm, M/L =  ± 6.4 to 8.7 mm) [21].

**Table 1:** Oligonucleotide primer sequences used for qRT-PCR

Gene	Primer sequence (forward)	Primer sequence (reverse)	Annealing temperature (°C)	Product size (bp)
*NFKBIA*	GAA AAT CTT CAG ACG CTG CCC	AGG GCA ACT CAT CTT CCG TG	60	81
*EGR1*	TCG CTC GGA TGA GCT TAC AC	CAA AAG GCT TCT CGC CTG TG	60	139
*MT1M*	CCA ACT GCT CCT GTG CCA	GCA GCT TTT CTT GCA GGA GG	60	91
*DUSP1*	GGG CAC CTC TAC TAC AAC GG	CTC GGA GAG GTT GTG ATG GG	60	104
*MT1A*	CCC AAC TGC TCC TGC TCC	ATT TGC AGT TCT TGC AGC CG	60	69
*FOS*	TAT TTT GGC AGC CCA CCG A	GCA GAC CCC CAG TCA AGT C	60	97
*MT2A*	CTG GCT CCT GCA AAT GCA AA	CAG ATG CAG CCC TGG GAG	60	102
*SLPI*	GTT CCC ATT CGT GGA CCA GT	CCC ACA CAT ACC CTC ACA ACA	60	140
*PER1*	CTG TGG GGG CCA AGA AAG AT	GGC TCC TTC CGA GGA GTT G	60	69
*COLEC10*	GAA ACC AGT TCA TCC TGC TGC	AGC AGA GCG ACC ATC AAC AT	60	76
*SYT9*	AAC TCT CGT GGT TAC CGC C	CCA GCA CAG TTT CCA AGA CAC	60	72
*SLITRK6*	CGG CTG GTA CCC TTT TGA GT	CGC GGC GCA GAA TAC AAT AG	60	100
*KCNJ16*	GGC GGC GTT TTT ATT CTC CC	TTC TTC CGT GAC ACA ACG GT	60	69
*SLC5A7*	ATC TAT GGA AAG CGC ATG GGT	TAC GCT GAT GGT AGC CCC TA	60	104
*CHRNA3*	TGT TCC AGT ACC TGT TCG AAG ATT	AGC CAC AGG TTG GTT TCC AT	60	147
*SLC4A5*	TAG AGG GTG GAC TTC TGC GA	AAT GTC TGC AAG GGA ACG GT	60	109
*SLC40A1*	GCA GCT GAC CTC ACC TAA AGA	AGG CAC AGG TGG GTT CTT G	60	115
*SLC5A3*	ACC TCC CAC GAA GGA TCA GA	AGA GCA ACT CTC CTT CGT CAC	60	76
*IGF2*	ATC TCT TTG GCC TTC GCC TT	CAG ACA AAC TGA AGC GTG TCA A	60	94
*KL*	GAC GGC ATC AAC CTT TGT GG	AAA AGC CGG ACT TGG GAA CT	60	69

*NFKBIA: nuclear factor of kappa light polypeptide gene enhancer in B-cells inhibitor, alpha; EGR1: early growth response 1; MT1M: metallothionein 1 M; DUSP1: dual specificity phosphatase 1; MT1A: metallothionein 1a; FOS: FBJ osteosarcoma oncogene; MT2A: metallothionein 2A; SLPI: secretory leukocyte peptidase inhibitor; PER1: period circadian clock 1; COLEC10: collectin subfamily member 10 (C-type lectin); SYT9: synaptotagmin IX; SLITRK6: SLIT and NTRK-like family, member 6; KCNJ16: potassium channel, inwardly rectifying subfamily J, member 16; SLC5A7: solute carrier family 5 (sodium/choline cotransporter), member 7; CHRNA3: cholinergic receptor, nicotinic, alpha 3; SLC4A5: solute carrier family 4, sodium bicarbonate cotransporter, member 5; SLC40A1: solute carrier family 40 (iron-regulated transporter), member 1; SLC5A3: solute carrier family 5 (sodium/myo-inositol cotransporter), member 3; IGF2: insulin-like growth factor 2; KL: Klotho*

### Statistical analysis

The ABR thresholds were compared between the noise and control groups using an independent sample T-test. The expression of each mRNA in the hippocampus and primary auditory cortex of the noise and control groups were compared using the Mann–Whitney U test because the data did not show a normal distribution in the Shapiro–Wilk test. The quantified data are presented as means ± standard deviations in graphs. SPSS software (ver. 21.0; IBM Corp., Armonk, NY, USA) was used for the analyses, and P ≤ 0.05 was deemed a statistically significant difference.

## Results

The mean hearing thresholds in the noise group were 61.33 dB SPL (standard deviation [SD] = 6.40), 90.00 dB SPL (SD = 0.00), 90.00 dB SPL (SD = 0.00), and 90.00 dB SPL (SD = 0.00) at 4, 8, 16, and 32 kHz, respectively. The control group had average hearing thresholds of 40.67 dB SPL (SD = 11.63), 36.67 dB SPL (SD = 4.88), 27.33 dB SPL (SD = 4.58), and 41.33 dB SPL (SD = 8.34) at 4, 8, 16, and 32 kHz, respectively.

In the microarray analyses, 38 annotated genes were significantly upregulated in the noise group compared to the control group (Additional file 1: Table S1). On the other hand, 81 annotated genes were significantly downregulated in the noise group compared to the control group (Additional file 1: Table S2). These 38 upregulated and 81 downregulated genes were used for pathway analyses of upregulated pathways and downregulated pathways, respectively.

The pathway analyses identified upregulated and downregulated pathways in the noise group (Table 2). The upregulated genes were related to cellular responses to external stimuli (4 involved genes, *P* < 0.001), cytokine signaling in the immune system (4 involved genes, *P* < 0.001), and the immune system (6 involved genes, *P* < 0.001). These upregulated pathways included the upregulated genes *collectin subfamily member 10 (C-type lectin) (COLEC10), period circadian regulator 1 (PER1), secretory leukocyte peptidase inhibitor (SLPI), metallothionein 2A (MT2A), metallothionein 1A (MT1A), FBJ osteosarcoma oncogene (FOS), nuclear factor of kappa light polypeptide gene enhancer in B-cell inhibitor, alpha (NFKBIA), metallothionein 1 M (MT1M), early growth response 1 (EGR1),* and *dual specificity phosphatase 1 (DUSP1)* (Table 3). On the other hand, the downregulated genes were associated with neuronal systems (5 involved genes, *P* < 0.001), solute carrier (SLC)-mediated transmembrane transport (4 involved genes, *P* < 0.001), transport of bile salts and organic acids (3 involved genes, *P* < 0.001), metal ions and amine compounds (3 involved genes, *P* < 0.001), transmission across chemical synapses (3 involved genes, *P* < 0.001), and signaling by type 1 insulin-like growth factor 1 receptor (2 involved genes, *P* < 0.001). These downregulated pathways included the downregulated genes *cholinergic receptor, nicotinic, alpha 3 (CHRNA3), insulin-like growth factor 2 (IGF2), potassium channel, inwardly rectifying subfamily J, member 16 (KCNJ16), Klotho (KL), solute carrier family 40 (iron-regulated transporter), member 1 (SLC40A1), solute carrier family 4, sodium bicarbonate cotransporter, member 5 (SLC4A5), solute carrier family 5 (sodium/myo-inositol cotransporter), member 3 (SLC5A3), solute carrier family 5 (sodium/choline cotransporter), member 7 (SLC5A7), SLIT and NTRK-like family, member 6 (SLITRK6),* and *synaptotagmin IX (SYT9)*.

**Table 2:** Pathways identified as upregulated and downregulated

Pathway name	Candidate mRNAs	*P* value	Q value	Pathway source
Upregulated pathways				
Cellular responses to external stimuli	4 (*MT1M, FOS, MT2A, MT1A*)	0.000181	0.00227	Reactome
Cytokine signaling in immune system	4 (*NFKBIA, FOS, MT2A, EGR1*)	0.00027	0.00264	Reactome
Immune system	6 (*NFKBIA, SLPI, EGR1, FOS, MT2A, COLEC10*)	0.000968	0.00452	Reactome
TRAF6-mediated induction of NFκB and MAP kinases upon TLR7/8 or 9 activation	2 (*NFKBIA, FOS*)	0.00197	0.00452	Reactome
Circadian entrainment	2 (*PER1, FOS*)	0.00234	0.00452	KEGG
TNF signaling pathway	2 (*EGR1, MT2A*)	0.00306	0.00463	KEGG
Relaxin signaling pathway	2 (*NFKBIA, FOS*)	0.00424	0.00589	KEGG
Apoptosis	2 (*NFKBIA, FOS*)	0.00463	0.00626	KEGG
MAPK signaling pathway	2 (*DUSP1, FOS*)	0.0205	0.0214	KEGG
Downregulated pathways				
Neuronal system	5 (*SYT9, SLITRK6, SLC5A7, CHRNA3, KCNJ16*)	0.00000392	0.0000549	Reactome
SLC-mediated transmembrane transport	4 (*SLC5A7, SLC40A1, SLC5A3, SLC4A5*)	0.0000233	0.000152	Reactome
Transport of bile salts and organic acids, metal ions and amine compounds	3 (*SLC5A7, SLC40A1, SLC5A3*)	0.0000325	0.000152	Reactome
Transmission across chemical synapses	3 (*CLS5A7, KCNJ16, CHRNA3*)	0.000548	0.00133	Reactome
Signaling by type 1 insulin-like growth factor 1 receptor (IGF1R)	2 (*IGF2, KL*)	0.000666	0.00133	Reactome
Protein–protein interactions at synapses	2 (*SYT9, SLITRK6*)	0.00197	0.00282	Reactome
Longevity regulating pathway	2 (*KL, IGF2*)	0.00201	0.00282	KEGG
Cholinergic synapse	2 (*SLC5A7, CHRNA3*)	0.00317	0.00403	KEGG
Neurotransmitter receptors and postsynaptic signal transmission	2 (*KCNJ16, CHRNA3*)	0.00575	0.00671	Reactome

**Table 3:** The differentially expressed genes included in the pathway analyses

	Gene Symbol	Description	Fold changes	*P* value
Upregulated genes	*COLEC10*	*collectin subfamily member 10 (C-type lectin)*	1.69	0.041
	*PER1*	*period circadian clock 1*	1.84	0.000
	*SLPI*	*secretory leukocyte peptidase inhibitor*	1.83	0.025
	*MT2A*	*metallothionein 2A*	2.19	0.027
	*MT1A*	*metallothionein 1A*	2.51	0.003
	*FOS*	*FBJ osteosarcoma oncogene*	3.19	0.001
	*NFKBIA*	*nuclear factor of kappa light polypeptide gene enhancer in B-cells inhibitor, alpha*	2.21	0.000
	*MT1M*	*metallothionein 1 M*	1.98	0.029
	*EGR1*	*early growth response 1*	1.53	0.019
	*DUSP1*	*dual specificity phosphatase 1*	2.64	0.001
Downregulated genes	*CHRNA3*	*cholinergic receptor, nicotinic, alpha 3*	2.26	0.049
	*IGF2*	*insulin-like growth factor 2*	1.74	0.030
	*KCNJ16*	*potassium channel, inwardly rectifying subfamily J, member 16*	1.54	0.020
	*KL*	*Klotho*	5.57	0.045
	*SLC40A1*	*solute carrier family 40 (iron-regulated transporter), member 1*	1.56	0.005
	*SLC4A5*	*solute carrier family 4, sodium bicarbonate cotransporter, member 5*	4.78	0.044
	*SLC5A3*	*solute carrier family 5 (sodium/myo-inositol cotransporter), member 3*	1.66	0.004
	*SLC5A7*	*solute carrier family 5 (sodium/choline cotransporter), member 7*	1.64	0.014
	*SLITRK6*	*SLIT and NTRK-like family, member 6*	2.21	0.035
	*SYT9*	*synaptotagmin IX*	1.73	0.044

qRT-PCR was performed to confirm the DE genes in these pathways. Among the upregulated genes, the expression of the *MT2A, MT1A, FOS, NFKBIA, MT1M*, and *DUSP1* mRNAs was increased in the noise group compared to the control group (3.00-fold (3.49/1.16) [SD = 0.58], *P* < 0.001 for *MT2A*, 2.96-fold (3.49/1.18) [SD = 0.58], *P* < 0.001 for *MT1A*, 5.01-fold (5.46/1.09) [SD = 0.41], *P* < 0.001 for *FOS*, 2.82-fold (4.48/1.59) [SD = 0.91], *P* = 0.006 for *NFKBIA*, 2.88-fold (4.20/1.46) [SD = 0.73], *P* = 0.002 for *MT1M*, and 2.66-fold (2.79/1.05) [SD = 0.46], *P* = 0.008 for *DUSP1*) (Fig. 3). Among the upregulated genes confirmed by RT-PCR, the *FOS* mRNA exhibited the greatest difference in expression, with 5.01-fold higher levels in the noise group than in the control group (95% confidence interval [95% CI] = 4.52–6.40). Among the downregulated genes, the expression of the *CHRNA3* [0.34-fold (0.29/0.85) (SD = 0.02)], *IGF2* [0.57-fold (0.39/0.69) (SD = 0.01)], *KCNKJ16* [0.74-fold (0.71/0.96) (SD = 0.03)], *KL* [0.08-fold (0.49/0.04) (SD = 0.01)], *SLC40A1* [0.58-fold (0.56/0.96) (SD = 0.10)], *SLC4A5* [0.27-fold (0.17/0.63) (SD = 0.02)], *SLC5A7* (0.30-fold (0.54/1.83) (SD = 0.24))] *SLITRK6* [0.34-fold (0.28/0.83) (SD = 0.03]and *SYT9* [0.56-fold (0.49/0.87) (SD = 0.02)] mRNAs were decreased in the noise group (*P* < 0.01 for all genes) (Fig. 4). Among the downregulated genes confirmed by RT-PCR, the *KL* mRNA exhibited lowest expression, with 0.08-fold higher levels in the noise group than in the control group (95% CI = 0.06–0.10).

The changes in the mRNA expression of the upregulated and downregulated genes, which were confirmed using qRT-PCR, in the temporal cortex were examined using qRT-PCR (Fig. 5). The expression of the *MT2A* [2.69-fold (2.32/0.86) (SD = 0.03), *P* = 0.002], *MT1A* [4.30-fold (5.50/1.28) (SD = 0.61), *P* = 0.002], *MT1M* [2.60-fold (2.52/0.97) (SD = 0.09), *P* = 0.002], and *NFKBIA* [4.89-fold (5.77/1.18) (SD = 0.91),* P* = 0.002] mRNAs were increased in the temporal cortex of the noise group. However, the expression of the *DUSP1* [1.41-fold (1.17/0.83) (SD = 0.14), *P* = 0.13] and *FOS* [0.85-fold (0.81/0.95) (SD = 0.19), *P* = 0.24] mRNAs in the temporal cortex was not significantly different between the noise and control groups. Regarding the genes that were downregulated in the hippocampus, the expression of the *SLC5A7* [0.31-fold (0.25/0.80) (SD = 0.05), *P* = 0.004] and *SLITRK6* [0.35-fold (0.35/1.00) (SD = 0.09), *P* = 0.004] mRNAs was decreased in the temporal cortex of the noise group. However, the levels of the *CHRNA3* [0.76-fold (0.76/1.00) (SD = 0.31],) *P* = 0.31], *KCNKJ16* [1.77-fold (2.25/1.27) (SD = 0.68), *P* = 0.59], *KL* [0.58-fold (0.47/0.81) (SD = 0.16), *P* = 0.24], *SLC40A1* [0.59-fold (0.59/1.00) (SD = 0.21), *P *= 0.13], *SLC4A5* [0.80-fold (0.80/1.00) (SD = 0.20), *P* = 0.39], and *SYT9* [1.34-fold (1.34/1.00) (SD = 0.13), *P* = 0.38] mRNAs in the temporal cortex were not significantly different between the noise and control groups. The expression of the *IGF2* mRNA was increased in the primary auditory cortex of the noise group [1.80-fold (1.80/1.00) (SD = 0.47), *P* = 0.003].

## Discussion

The present study examined changes in gene expression in the hippocampus after noise exposure. The sufficiently intense noise stimuli induced large hearing threshold shifts, as evidenced by changes in the ABR thresholds. Acute traumatic exposure to noise increased the expression of genes related to the inflammatory response, immune system, and apoptosis and decreased the expression of genes associated with neurotransmission and synapses in the hippocampus. In particular, the greatest changes in expression were observed for the *FOS* and *KL* mRNAs in the hippocampus following noise exposure. *FOS* expression was increased, while *KL* expression was reduced in the noise group. To our knowledge, no previous study has conducted a microarray analysis of the hippocampus after noise exposure.

Genes related to neurotransmission and synapses were downregulated after noise exposure in the present study. Consistent with the results of the present study, several previous studies have reported the suppression of neurogenesis in the hippocampus following noise exposure [22, 23]. Moreover, although data from gene expression microarrays of the hippocampus after noise exposure are lacking, a number of previous studies have reported changes in gene expression after exposure to other types of stress. Acute restraint stress upregulated the expression of genes related to neurogenesis and neuronal protection, including *Ttr, Rab6, Gh, Prl, Ndufb9* and *Ndufa6*, in mice [24]. This upregulation of genes related to neurogenesis and neuronal protection might be a compensatory response to acute stress. On the other hand, chronic restraint stress for 4 weeks reduced neurogenesis by downregulating the expression of the brain-specific transcription factor neuronal PAS domain protein 4 [25]. Likewise, the downregulation of genes involved in neurogenesis and synaptic factors was observed in mice after 7 days of restraint stress [26]. Therefore, we presumed that long-term or intense stress impairs neurogenesis and induces neuronal injury.

Among the downregulated genes identified in the noise group, the expression of *KL* exhibited the greatest decrease in the present study. *KL* encodes a type I single-pass transmembrane protein [27] that is secreted into the extracellular space, cerebrospinal fluid, and blood serum. *KL* is expressed at high levels in the choroid plexus in the lateral ventricle and is detected throughout the brain, including the hippocampus [28]. *KL* has been reported to be associated with aging and Alzheimer’s disease [29]. Several putative mechanisms have been suggested for the anti-aging and neuroprotective effects of KL. KL affects the Akt and ERK signaling pathways and thereby promotes the maturation of primary oligodendrocytic progenitor cells and myelination in a study using knockout mice [30]. In addition, KL increases erythropoietin (Epo) receptor expression, which promoted Jak2 and Stat5 phosphorylation and provides neuroprotection from peroxide-induced cytotoxicity in vitro [31]. *KL* knockout mice show elevated levels of proinflammatory factors and macrophage infiltration in the choroid plexus [32]. Overexpression of the secreted form of KL in the hippocampus reverses aging-related effects and cognitive dysfunction in mice [33]. Thus, KL potentially represents a protective or therapeutic target for hippocampal or cognitive dysfunction following noise-induced hearing loss.

Genes involved in the cellular responses to external stimuli and the immune system were upregulated after noise exposure in the present study. A study with restrained rats reported the upregulation of genes involved in the responses to stimuli and extracellular matrix receptor interaction pathways in the hippocampus [34]. However, genes related to the immune system process were downregulated in that study [34], in contrast to the results in the present study. This discrepancy might be due to the chronicity and types of stress stimuli, which persisted for more than 7 days in the study of rats subjected to restraint stress. The immune system might be suppressed after chronic stress, while it is potentially activated in the acute phase of stress exposure. In the present study, NFκB pathway-related genes, including *NFKBIA*, were upregulated in the noise group. Similarly, NFκB pathway genes, including *NFKBIA, RELA* and *NFKB1,* were upregulated after restraint stress in mice [35].

Interestingly, *FOS* expression exhibited the greatest increase following noise exposure in this study. *FOS* is an immediate-early gene, and its expression is increased following stress exposure [36]. Under stress conditions, *FOS* expression is induced by the increased level of glucocorticoid hormones, which have been shown to activate the cascades of extracellular signal-regulated kinase mitogen-activated protein kinase (ERK/MAPK) signaling involving ERK1/2, mitogen- and stress-activated kinase 1 (MSK1), and ETS domain protein-1 (ELK1) [37]. However, the corticosterone level was not increased until 2 weeks after noise exposure and gradually decreased below the control levels at 2–10 weeks after noise exposure [38]. Thus, the stress hormone response might not be the sole putative mechanism responsible for hippocampal changes following noise exposure. In addition to the stress component due to noise exposure, auditory deprivation might affect the changes in the hippocampus observed in the present study. In support of this hypothesis, the primary temporal cortex and the hippocampus exhibited changes in gene expression after acute noise exposure in the present study. Auditory processing has been suggested to be modulated by the hippocampal circuit and the central auditory pathways [39]. Dysregulation of the hippocampal circuit connected to the auditory system was suggested to be related to tinnitus and hyperacusis [40]. In addition, the hippocampus is even more vulnerable to noise exposure than the auditory cortex [41]. Indeed, not all the upregulated or downregulated genes in the hippocampus exhibited changes in mRNA expression in the primary auditory cortex in the present study. The expression of the *FOS* and *KL* mRNAs was not altered in the primary auditory cortex in the noise group in the present study. The decrease in neurotransmission and synapses might be attributed to the decreased activity of the auditory processing circuit in the hippocampus.

Although behavioral changes were not examined in this study, a previous study reported behavioral changes in habituation memory and exploratory activity, anxiety-related behavior, and the memory of an aversive experience, all of which are known to depend on the hippocampus [11]. The acute noise-induced hearing loss model minimized confounding factors due to other environmental factors, such as aging, and revealed the early effects of noise on changes in gene expression in the hippocampus. However, although all rats were housed under identical standard conditions, we were unable to exclude the possible heterogeneous response to noise exposure in this study. Moreover, temporal changes in both upregulated and downregulated genes in the hippocampus according to the duration of deafness warrant further study. In addition, the cell types and changes in protein expression were not specifically determined in this study. Further studies are warranted to resolve the limitations of the present study.

## Conclusions

Acute noise exposure induced changes in gene expression in the hippocampus. Genes associated with the immune system, including *FOS*, and the cellular response to external stimuli were upregulated after noise exposure. On the other hand, genes related to neurotransmission and synapses, such as *KL*, were downregulated following noise exposure.

## Supplementary information

**Additional file 1:** **Table S1.** Genes that were upregulated by noise exposure by at least 1.5-fold with *P*≤0.05 in microarray analyses of the hippocampus. **Table S2.** Genes that were downregulated by noise exposure by at least 1.5-fold with *P*≤0.05 in microarray analyses of the hippocampus.

## Data Availability

The data used in our study are available from the authors upon reasonable request. The data were deposited in the GEO database (GSE140087, https://www.ncbi.nlm.nih.gov/geo/query/acc.cgi?acc=GSE140087).

## Abbreviations

qRT-PCR: Quantitative reverse transcription-polymerase chain reaction
COLEC10: Collectin subfamily member 10 (C-type lectin)
PER1: Period circadian regulator 1
SLPI: Secretory leukocyte peptidase inhibitor
MT2A: Metallothionein 2A
MT1A: Metallothionein 1A
FOS: FBJ osteosarcoma oncogene
NFKBIA: Nuclear factor of kappa light polypeptide gene enhancer in B-cell inhibitor, alpha
MT1M: Metallothionein 1 M
EGR1: Early growth response 1
DUSP1: Dual specificity phosphatase 1
CHRNA3: Cholinergic receptor, nicotinic, alpha 3
IGF2: Insulin-like growth factor 2
KCNJ16: Potassium channel, inwardly rectifying channel, subfamily J, member 16
KL: Klotho
SLC40A1: Solute carrier family 40 (iron-regulated transporter), member 1
SLC4A5: Solute carrier family 4, sodium bicarbonate cotransporter, member 5
SLC5A7: Solute carrier family 5 (sodium/choline cotransporter), member 7
SLITRK6: SLIT and NTRK-like family, member 6
SYT9: Synaptotagmin IX
SLC5A3: Solute carrier family 5 (sodium/myo-inositol cotransporter), member 3
MSK1: Mitogen- and stress-activated kinase 1
ELK1: ETS domain protein-1
ABRs: Auditory brainstem responses
CA3: Cornu ammonis 3
DE: Differentially expressed
SD: Standard deviation
