# Supplementary material for: Effect of acute noise trauma on the gene expression profile of the hippocampus
Source: BMC Neurosci. 2020 Nov 7;21:45. doi: 10.1186/s12868-020-00599-9 (PMC7648995; doi:10.1186/s12868-020-00599-9)
Supplement: Supplementary file 1 — Additional file 1: Table S1. Genes that were upregulated by noise exposure by at least 1.5-fold with P≤0.05 in microarray analyses of the hippocampus. Table S2. Genes that were downregulated by noise exposure by at least 1.5-fold with P≤0.05 in microarray analyses of the hippocampus. [file 12868_2020_599_MOESM1_ESM.docx]

**Table S1** Genes that were upregulated by noise exposure by at least 1.5-fold with P ≤ 0.05 in microarray analyses of the hippocampus

| **Gene Symbol** | **Description** | **Fold change** | **P-value** |
| --- | --- | --- | --- |
| *FOS* | *FBJ osteosarcoma oncogene* | 3.19 | 0.001 |
| *CIART* | *circadian associated repressor of transcription* | 3.00 | 0.000 |
| *ZFP36* | *zinc finger protein 36* | 2.75 | 0.000 |
| *DUSP1* | *dual specificity phosphatase 1* | 2.64 | 0.001 |
| *MT1A* | *metallothionein 1a* | 2.51 | 0.003 |
| *NFKBIA* | *nuclear factor of kappa light polypeptide gene enhancer in B-cells inhibitor, alpha* | 2.21 | 0.000 |
| *MIRLET7C-2* | *microRNA let7c-2* | 2.20 | 0.049 |
| *MT2A* | *metallothionein 2A* | 2.19 | 0.027 |
| *APOLD1* | *apolipoprotein L domain containing 1* | 1.98 | 0.004 |
| *MT1M* | *metallothionein 1M* | 1.98 | 0.029 |
| *LOC100911408* | *nidogen-2-like* | 1.86 | 0.024 |
| *OLR137* | *olfactory receptor 137* | 1.85 | 0.041 |
| *PER1* | *period circadian clock 1* | 1.84 | 0.000 |
| *HIST1H2AF* | *histone cluster 1, H2af* | 1.83 | 0.004 |
| *SLPI* | *secretory leukocyte peptidase inhibitor* | 1.83 | 0.025 |
| *LOC684681* | *similar to Histone H1.2 (H1 VAR.1) (H1c)* | 1.81 | 0.003 |
| *OLR1236* | *olfactory receptor 1236* | 1.71 | 0.026 |
| *COLEC10* | *collectin sub-family member 10 (C-type lectin)* | 1.69 | 0.041 |
| *VOM1R92* | *vomeronasal 1 receptor 92* | 1.67 | 0.015 |
| *LOC102552542; LOC102550512; LOC102550416* | *small proline-rich protein 2D-like; small proline-rich protein 2I-like [Source:RGD Symbol;Acc:7654558]* | 1.67 | 0.007 |
| *OLR1406* | *olfactory receptor 1406* | 1.67 | 0.043 |
| *RCCD1* | *RCC1 domain containing 1* | 1.64 | 0.013 |
| *OLR220* | *olfactory receptor 220* | 1.64 | 0.007 |
| *KRT81* | *keratin 81, type II* | 1.64 | 0.031 |
| *MIR382* | *microRNA 382* | 1.63 | 0.006 |
| *OAS1B* | *2-5 oligoadenylate synthetase 1B* | 1.63 | 0.025 |
| *KLRC2; KLRC3* | *killer cell lectin-like receptor subfamily C, member 2; killer cell lectin-like receptor subfamily C, member 3* | 1.61 | 0.020 |
| *CSRNP1* | *cysteine-serine-rich nuclear protein 1* | 1.60 | 0.050 |
| *OLR346* | *olfactory receptor 346* | 1.60 | 0.041 |
| *SLC7A12* | *solute carrier family 7 (cationic amino acid transporter, y+ system), member 12* | 1.58 | 0.034 |
| *OLR329* | *olfactory receptor 329* | 1.58 | 0.035 |
| *RGD1566184* | *similar to Testis derived transcript* | 1.58 | 0.041 |
| *LOC690655* | *similar to High mobility group protein 1 (HMG-1) (High mobility group protein B1) (Amphoterin) (Heparin-binding protein p30)* | 1.57 | 0.006 |
| *OLR1104* | *olfactory receptor 1104* | 1.56 | 0.004 |
| *ARRDC4* | *arrestin domain containing 4* | 1.55 | 0.002 |
| *SGK1* | *serum/glucocorticoid regulated kinase 1* | 1.54 | 0.020 |
| *LOC685699* | *hypothetical protein LOC685699* | 1.54 | 0.018 |
| *EGR1* | *early growth response 1* | 1.53 | 0.019 |

**Table S2** Genes that were downregulated by noise exposure by at least 1.5-fold with P ≤ 0.05 in microarray analyses of the hippocampus

| **Gene Symbol** | **Description** | **Fold change** | **P-value** |
| --- | --- | --- | --- |
| *TTR* | *transthyretin* | 19.17 | 0.015 |
| *KL* | *Klotho* | 5.57 | 0.045 |
| *KCNJ13* | *potassium channel, inwardly rectifying subfamily J, member 13* | 5.12 | 0.033 |
| *SLC4A5* | *solute carrier family 4, sodium bicarbonate cotransporter, member 5* | 4.78 | 0.044 |
| *MIR384* | *microRNA 384* | 4.48 | 0.000 |
| *LOC102555051* | *zinc finger protein 709-like* | 2.52 | 0.005 |
| *RGD1359158* | *similar to RIKEN cDNA 1110059E24* | 2.39 | 0.046 |
| *LOC102553278* | *zinc finger protein 709-like* | 2.26 | 0.003 |
| *CHRNA3* | *cholinergic receptor, nicotinic, alpha 3* | 2.26 | 0.049 |
| *CLK1* | *CDC-like kinase 1* | 2.22 | 0.002 |
| *SLITRK6* | *SLIT and NTRK-like family, member 6* | 2.21 | 0.035 |
| *CCR5* | *chemokine (C-C motif) receptor 5* | 2.19 | 0.002 |
| *HMGN5* | *high mobility group nucleosome binding domain 5* | 2.14 | 0.011 |
| *LOC654482* | *hypothetical protein LOC654482* | 2.14 | 0.016 |
| *ZIC1* | *Zic family member 1* | 2.13 | 0.016 |
| *ZFP248* | *zinc finger protein 248* | 2.02 | 0.006 |
| *CLDN1* | *claudin 1* | 1.98 | 0.033 |
| *LOC100912582* | *zinc finger protein 14-like* | 1.95 | 0.031 |
| *CCDC141* | *coiled-coil domain containing 141* | 1.91 | 0.011 |
| *FMOD* | *fibromodulin* | 1.90 | 0.024 |
| *ZFP945* | *zinc finger protein 945* | 1.90 | 0.047 |
| *GPR151* | *G protein-coupled receptor 151* | 1.88 | 0.001 |
| *CWC22* | *CWC22 spliceosome-associated protein* | 1.86 | 0.049 |
| *LOC102550291* | *zinc finger protein 658-like* | 1.82 | 0.039 |
| *TLR3* | *toll-like receptor 3* | 1.82 | 0.003 |
| *CD244* | *Cd244 molecule, natural killer cell receptor 2B4* | 1.82 | 0.012 |
| *RGD1563958* | *similar to 60S ribosomal protein L32 [Source:RGD Symbol;Acc:1563958]* | 1.76 | 0.038 |
| *CTS8L1* | *cathepsin 8-like 1* | 1.76 | 0.027 |
| *SOX18* | *SRY (sex determining region Y)-box 18* | 1.75 | 0.011 |
| *IGF2* | *insulin-like growth factor 2* | 1.74 | 0.030 |
| *MTG2* | *mitochondrial ribosome-associated GTPase 2* | 1.74 | 0.000 |
| *SYT9* | *synaptotagmin IX* | 1.73 | 0.044 |
| *LEF1* | *lymphoid enhancer binding factor 1* | 1.72 | 0.010 |
| *WDR89* | *WD repeat domain 89* | 1.72 | 0.006 |
| *EVI2B* | *ecotropic viral integration site 2B* | 1.71 | 0.008 |
| *RSL1* | *regulator of sex limited protein 1* | 1.70 | 0.000 |
| *FAM84B* | *family with sequence similarity 84, member B* | 1.70 | 0.022 |
| *ZFP667* | *zinc finger protein 667* | 1.70 | 0.010 |
| *RBMXL1* | *RNA binding motif protein, X-linked-like 1* | 1.69 | 0.008 |
| *PHACTR2* | *phosphatase and actin regulator 2* | 1.68 | 0.028 |
| *LCE1F* | *late cornified envelope 1F* | 1.68 | 0.010 |
| *SLC5A3* | *solute carrier family 5 (sodium/myo-inositol cotransporter), member 3* | 1.66 | 0.004 |
| *MIR501* | *microRNA 501* | 1.66 | 0.004 |
| *NT5C3A* | *5-nucleotidase, cytosolic IIIA* | 1.66 | 0.018 |
| *OLR1382* | *olfactory receptor 1382* | 1.65 | 0.029 |
| *MIR370* | *microRNA 370* | 1.65 | 0.042 |
| *ZFP52* | *zinc finger protein 52* | 1.64 | 0.027 |
| *KBTBD3* | *kelch repeat and BTB (POZ) domain containing 3* | 1.64 | 0.014 |
| *SLC5A7* | *solute carrier family 5 (sodium/choline cotransporter), member 7* | 1.64 | 0.014 |
| *FAM46A* | *family with sequence similarity 46, member A* | 1.63 | 0.030 |
| *ZFP53* | *zinc finger protein 53* | 1.62 | 0.018 |
| *PTCHD1* | *patched domain containing 1 [Source:RGD Symbol;Acc:1564527]* | 1.62 | 0.025 |
| *MCF2* | *MCF.2 cell line derived transforming sequence; MCF.2 cell line derived transforming sequence [Source:RGD Symbol;Acc:1566098]* | 1.62 | 0.042 |
| *NPAS4* | *neuronal PAS domain protein 4* | 1.61 | 0.015 |
| *MDFIC* | *MyoD family inhibitor domain containing* | 1.61 | 0.039 |
| *ZIC5* | *Zic family member 5* | 1.61 | 0.036 |
| *XPA* | *xeroderma pigmentosum, complementation group A* | 1.61 | 0.001 |
| *LOC102556967* | *zinc finger protein 484-like* | 1.60 | 0.033 |
| *ANG2* | *angiogenin, ribonuclease A family, member 2* | 1.59 | 0.009 |
| *SOD3* | *superoxide dismutase 3, extracellular* | 1.59 | 0.011 |
| *LOC683430; LOC681355* | *similar to potassium channel tetramerisation domain containing 12b* | 1.58 | 0.001 |
| *NUDT13* | *nudix (nucleoside diphosphate linked moiety X)-type motif 13* | 1.58 | 0.002 |
| *SIMC1* | *SUMO-interacting motifs containing 1* | 1.58 | 0.003 |
| *OLR858* | *olfactory receptor 858* | 1.58 | 0.004 |
| *SLC40A1* | *solute carrier family 40 (iron-regulated transporter), member 1* | 1.56 | 0.005 |
| *ZFP780B-PS1* | *zinc finger protein 780B, pseudogene 1* | 1.55 | 0.005 |
| *ZFP27* | *zinc finger protein 27* | 1.54 | 0.012 |
| *FAM84B* | *family with sequence similarity 84, member B* | 1.54 | 0.018 |
| *KCNJ16* | *potassium channel, inwardly rectifying subfamily J, member 16* | 1.54 | 0.020 |
| *WDR78* | *WD repeat domain 78* | 1.54 | 0.009 |
| *NAPEPLD* | *N-acyl phosphatidylethanolamine phospholipase D* | 1.54 | 0.036 |
| *KRCC1* | *lysine-rich coiled-coil 1* | 1.54 | 0.003 |
| *WSB1* | *WD repeat and SOCS box-containing 1* | 1.54 | 0.001 |
| *GNPDA2* | *glucosamine-6-phosphate deaminase 2* | 1.54 | 0.023 |
| *PDGFD* | *platelet-derived growth factor D* | 1.53 | 0.011 |
| *PANK2* | *pantothenate kinase 2* | 1.53 | 0.021 |
| *MGC108823; LOC102549198; LOC100910979* | *similar to interferon-inducible GTPase; interferon-inducible GTPase 1-like* | 1.51 | 0.033 |
| *LOC501426; LOC103692727* | *putative V-set and immunoglobulin domain-containing-like protein IGHV4OR15-8* | 1.51 | 0.012 |
| *EFCAB7* | *EF-hand calcium binding domain 7* | 1.51 | 0.026 |
| *MOSPD2* | *motile sperm domain containing 2* | 1.51 | 0.024 |
| *LOC103691238* | *zinc finger protein 239-like* | 1.50 | 0.010 |
